# Lysophosphatidic Acid Stimulates MCP-1 Secretion from C2C12 Myoblast

**DOI:** 10.5402/2012/983420

**Published:** 2012-11-25

**Authors:** Tamotsu Tsukahara, Hisao Haniu

**Affiliations:** ^1^Department of Integrative Physiology and Bio-System Control, Shinshu University School of Medicine, 3-1-1 Asahi, Matsumoto, Nagano 390-8621, Japan; ^2^Department of Orthopaedic Surgery, Shinshu University School of Medicine, 3-1-1 Asahi, Matsumoto, Nagano 390-8621, Japan

## Abstract

Chemokines are regulatory proteins that play an important role in muscle cell migration and proliferation. In this study, C2C12 cells treated with lysophosphatidic acid (LPA) showed an increase in endogenous monocyte chemotactic protein-1 (MCP-1) expression and secretion. LPA is a naturally occurring bioactive lysophospholipid with hormone- and growth-factor-like activities. LPA is produced by activated platelets, cytokine-stimulated leukocytes, and possibly by other cell types. However, the LPA analog cyclic phosphatidic acid (cPA) had no effect on the expression and secretion of MCP-1. LPA, although similar in structure to cPA, had potent inducing effects on MCP-1 expression in C2C12 cells. In this study, we showed that LPA enhanced MCP-1 mRNA expression and protein secretion in a dose-dependent manner. Taken together, these results suggest that LPA enhances MCP-1 secretion in C2C12 cells and thus may play an important role in cell proliferation.

## 1. Introduction

Chemokines are a large family of structurally related proteins that play an essential role in leukocyte migration and differentiation [1]. They exert different functions under physiological conditions such as development and tissue repair [2]. Chemokines are small soluble molecules that are important in the communication between different cells [3, 4]. In recent years, significant advances have been made in understanding the molecular mechanisms involved in the regulation of skeletal muscle cell proliferation. Muscle cells are involved as targets in different pathological conditions such as autoimmune muscle disorders [5, 6]. Skeletal muscle cells are capable of producing and releasing a variety of molecules, including cytokines and chemokines [7]. Recently, chemokines have been considered to play important roles in skeletal muscle regeneration [8]. In response to a number of stimuli, myoblasts become activated, start to proliferate, and differentiate into multinucleated myotubes [9]. In the present study, we used a cytokine protein array to screen for the induction of different cytokines and chemokines. LPA, although similar in structure to cPA, had potent inducing effects on Monocyte chemotactic protein 1 (MCP-1) expression in C2C12 cells. MCP-1 is a chemokine that recruits monocytes to areas of vessel injury [10]. MCP-1 is a relatively basic protein of 8.7 kDa and is a heparin-binding C–C chemokine produced by monocytes. It is expressed in various tissues, including endothelial, bronchial, epithelial, and smooth muscle cells [11]. To identify MCP-1 that play a role in myogenesis, we examined the expression profiles of MCP-1 mRNA and protein. 

Lysophosphatidic acid (LPA) is a naturally occurring simple phospholipid that functions as a bioactive lipid mediator and a second messenger [12, 13]. It consists of a glycerol backbone with a hydroxyl group, a phosphate group, and a long-chain saturated or unsaturated fatty acid. LPA has been detected in biological fluids, and it exerts a wide variety of biological actions in cell proliferation, migration, morphological changes, and survival [13]. LPA is produced in serum after the activation of multiple biochemical pathways linked to platelet activation [14]. The concentration of LPA in the plasma is in the nanomolar range, whereas it can reach concentrations as high as 10 *μ*M in the serum during blood clotting [15]. LPA has attracted considerable interest because of its multiple roles in physiological and pathological conditions. LPA at low nanomolar concentrations activates monocytes [16] and recruits them by inducing IL-8 and MCP-1 expression in endothelial cells [17]. However, to date, no studies have examined whether LPA can promote production of cytokines or chemokines from C2C12 myoblasts. Although the chemotactic properties of MCP-1 are well recognized, we sought to investigate its influence on myoblast proliferation. To the best of our knowledge, 4 studies have examined the effect of C2C12 cell proliferation in vitro. Our study is the first to report that LPA induces MCP-1 secretion and increased cell proliferation in vitro. 

## 2. Materials and Methods

### 2.1. Reagents

LPA (18 : 1) and cyclic phosphatidic acid (cPA 18 : 1) were purchased from Avanti Polar Lipids (Alabaster, AL). Anti-MCP1 rabbit polyclonal antibody (sc-1304), MCP-1 small interfering RNA (siRNA) (sc-43914), and anti-*β*-actin mouse monoclonal antibody (sc-47778) were purchased from Santa Cruz Biotechnology Inc. (Santa Cruz, CA). 

### 2.2. Cell Culture

Mouse myogenic C2C12 cells were purchased from ECACC (European Collection of Cell Cultures). Cells were maintained in Dulbecco's modified Eagle's medium containing 10% fetal bovine serum (Sigma-Aldrich), penicillin (100 U/mL), and streptomycin (100 *μ*g/mL). Cells were grown and maintained in 100 mm culture plates (Iwaki, Tokyo, Japan) at 37°C in a 5% CO_2_ incubator. 

### 2.3. Measurement of Cytokine Concentrations in C2C12 Cell Culture Supernatant

The Q-Plex mouse cytokine array screen (Quansys Biosciences, Logan, UT) was used, according to the manufacturer's instructions, to measure the concentration of 16 cytokines and chemokines from culture supernatant. Thirty microliters of cytokine and chemokine standard or cell culture supernatant was added into the appropriate wells. After 1 h of incubation, the wells were washed 3 times and probed with streptavidin-HRP for 15 min at room temperature. The wells were again washed 3 times and the substrate was added; images were captured with a charge-coupled device (CCD) camera (ChemiDoc XRS, Bio-Rad Laboratories, Hercules, CA) and analyzed with the Quansys image analysis software. Cytokine and chemokine concentrations were calculated, and they were plotted using a standard curve. 

### 2.4. Western Blot Analysis

Cells were seeded onto 6-well plates (Iwaki, Tokyo, Japan) at a density of 1 × 10^5^ cells per well. After the indicated treatment, cells were lysed on ice for 30 min in a cell lysis buffer (20 mM Tris-HCl [pH 7.4], 10% [v/v] glycerol, 100 mM NaCl, 1% [v/v] Triton X-100, 1/100 protease inhibitor cocktail [Sigma], 1 mM dithiothreitol) and centrifuged at 16,000 ×g for 20 min at 4°C. The supernatants were collected as cell lysates and assayed for protein content by the Bradford method, using the Bio-Rad Protein Assay Kit. Cell lysates were then separated on 5–20% sodium dodecyl sulfate (SDS) polyacrylamide gels (e-PAGEL; ATTO, Tokyo, Japan) and electrotransferred onto Immobilon-P membranes (Millipore, Billerica, MA). The membranes were blocked with Block Ace (DS Parma Biomedical Co. Ltd., Osaka, Japan) for 1 h and incubated with a primary antibody in TBS-T with 5% Block Ace for 12 h at 4°C. After washing, the membrane was incubated with the secondary anti-rabbit IgG, horseradish peroxidase-linked species-specific whole antibody from a donkey (GE Healthcare, Little Chalfont, UK) for one hour at room temperature, and then visualized with EzWestLumi plus (ATTO). 

### 2.5. Quantitative Real-Time PCR Analysis

Total RNA was prepared using NucleoSpin RNA II (Takara, Shiga, Japan) from C2C12 cells. Total RNA (0.5 *μ*g) was used for the subsequent synthesis of cDNA with the ReverTra Ace qPCR RT Kit (Toyobo, Osaka, Japan) as recommended by the manufacturer. The mRNA levels were measured with an ECO Real-Time PCR system (Illumina Inc., San Diego, CA) and SYBR Green Realtime PCR Master Mix Plus (Toyobo) with the following primer pairs: mMCP-1, 5′-ATCCCAATGAGTAGGCTGGAGAGC-3′ (F) and 5′-CAGAAGTGCTTGAGGTGGTTGTG-3′ (R); 18S rRNA, 5′-CAGCCACCCGAGATTGAGCA-3′ (F) and 5′-TAGTAGCGACGGGCGGTGTG-3′ (R). All PCRs were performed in a 10 *μ*L volume, using 48-well PCR plates (Illumina). The cycling conditions were 95°C for 10 min (enzyme activation) followed by 40 cycles of 95°C for 15 s, 55°C for 15 s, and 72°C for 30 s. After amplification, the samples were slowly heated from 55°C to 95°C with continuous reading of fluorescence to obtain a melting curve. The relative mRNA level was calculated using the arithmetic formula 2^−ΔΔCq^, where ΔCq is the difference between the threshold cycle of a given target cDNA and that of an endogenous reference cDNA. Derivation of the formulas and validation tests has been described in Applied Biosystems User Bulletin No. 2.

### 2.6. Small Interfering RNA

C2C12 cells were seeded in 6-well culture plates (1 × 10^5^ cells per well) and incubated for 24 h. MCP-1 expression was inhibited in C2C12 cells by transfection with siRNA targeting MCP-1, using Lipofectamine RNAiMAX (Invitrogen). The reduction of MCP-1 levels was confirmed by western blot analysis.

### 2.7. Measurement of Cell Proliferation

C2C12 cells were seeded in 96-well culture plates (5 × 10^3^ cells per well) and incubated for 24 h. Cells were treated for 20 h with 10 *μ*M LPA (18 : 1) or cPA (18 : 1). Cell proliferation was determined with Cell Counting Kit-8 (Dojindo, Kumamoto, Japan). Ten microliters of Cell Counting Kit-8 was added to the medium, and cells were incubated for 2 h in a 5% CO_2_ incubator. The orange formazan dye generated was measured by determining the absorbance at 450 nm in a microplate reader (Awareness Technology Inc., Palm City, FL). 

### 2.8. Statistical Analysis

Statistical significance (*P* < 0.05) was determined by one-way ANOVA (for two groups) or Student's *t*-test (for independent groups).

## 3. Results and Discussion

**Figure 1:**
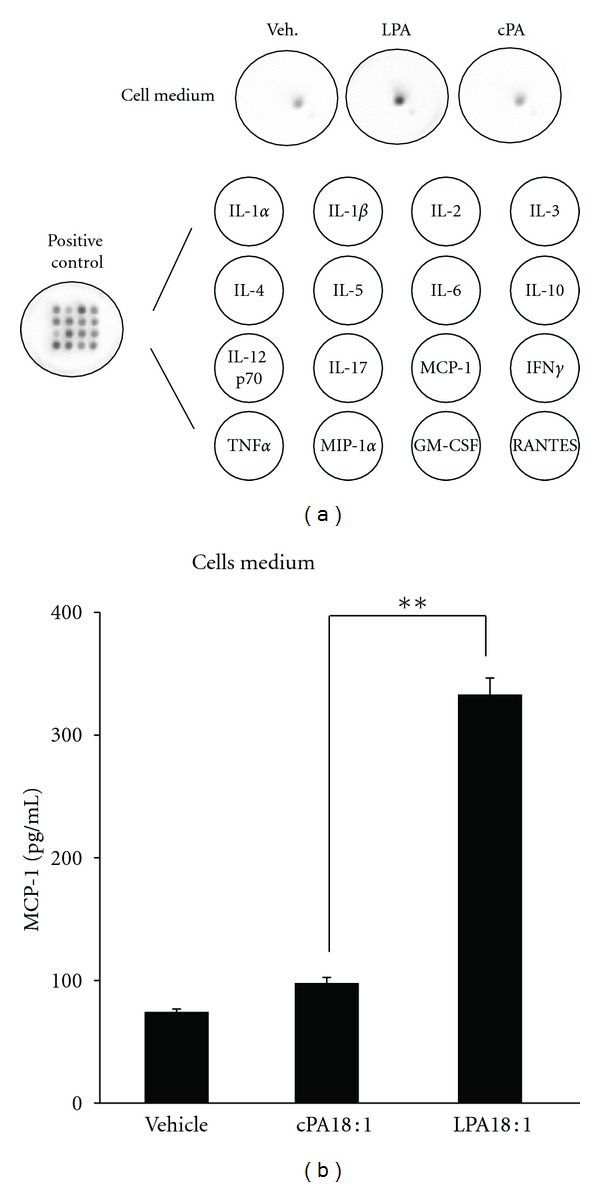
LPA induces MCP-1 production in C2C12 cell culture medium. (a) The culture supernatants were collected after 24 h of incubation with DMSO (vehicle), unsaturated forms of LPA (18 : 1) (10 *μ*M), and cPA (18 : 1) (10 *μ*M). The cytokine and chemokine levels in the supernatant were analyzed by Q-Plex cytokine array. The cytokine array included IL-1, IL-1, IL-2, IL-3, IL-4, IL-5, IL-6, IL-9, IL-10, IL-12, MCP-1, TNF-*α*, MIP-1, GM-CSF, and RANTES. (b) The images were captured with a CCD camera, and they were analyzed using the Quansys image analysis software. MCP-1 concentrations were calculated and plotted using a standard curve (mean ± SEM, *n* = 3; **P < 0.01 by ANOVA test).

A major finding in this study is that LPA activation in C2C12 cells may enhance MCP-1 expression and secretion. Constitutive chemokine expression has been reported in myoblast cell cultures [11], pointing to a significant role of chemokines in normal skeletal muscle physiology. As shown in Figure 1(a), MCP-1 was found to be constitutively expressed in cultured cells. To detect cytokines and chemokines secreted by C2C12 cells after exposure to LPA, we measured the levels of 16 cytokines and chemokines and compared them with those in vehicle control. Serum-starved C2C12 cells were treated or untreated with LPA, and conditioned media were collected and analyzed by an array assay. As shown in Figure 1(b), MCP-1 levels were significantly increased compared with the control after exposure to LPA for 24 h. Our results showed that LPA markedly induced the secretion of MCP-1 in the conditioned medium of C2C12 cells. LPA induction of MCP-1 in C2C12 cells has not been previously reported. Interestingly, other cytokines and chemokines that were probed for but not detected in these studies included IL-1, IL-1, IL-2, IL-3, IL-4, IL-5, IL-6, IL-9, IL-10, IL-12, TNF-*α*, MIP-1, GM-CSF, and RANTES. Furthermore, the LPA analog cPA showed no effect on MCP-1 release from C2C12 cells. Our cytokine and chemokine array results provide the first evidence that LPA induces MCP-1 secretion from C2C12 myoblasts. Although the pathway by which LPA, but not cPA, activates MCP-1 secretion is still unknown, these results suggest that LPA triggers the release of the chemotactic factor MCP-1 in C2C12 cells. The pathway through which the LPA receptor exerts its effects should be investigated in future trials. 

**Figure 2:**
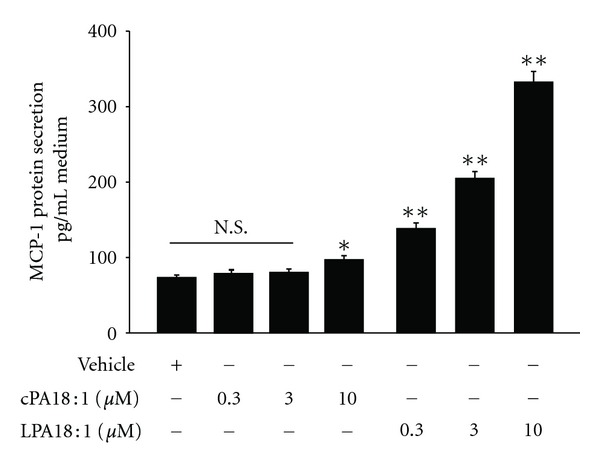
LPA dose dependently increased the MCP-1 release from C2C12 cells. The culture supernatants were collected after 24 h of incubation with DMSO (vehicle), LPA (18 : 1) (0.3, 3, and 10 *μ*M), and cPA (18 : 1) (0.3, 3, and 10 *μ*M). The cytokine and chemokine levels in the supernatant were analyzed by Q-Plex cytokine array. The images were captured with a CCD camera and analyzed using the Quansys image analysis software. MCP-1 concentrations were calculated and plotted using a standard curve (mean ± SEM, *n* = 3; **P < 0.01 by ANOVA test).

The MCP-1 level in the plasma has been considered as a marker for joint inflammation in rheumatoid arthritis (RA) [18]. The role of LPA in autoimmune diseases, including RA, has been reported [19]. As described in the previous section, accumulating evidence suggests that LPA promotes inflammation [17]. To better understand the LPA-mediated MCP-1 induction, we examined the dose-dependent effects of LPA (0.3–10 *μ*M) in a conditioned medium of C2C12 cells (Figure 2). Interestingly, LPA, but not cPA, increased the MCP-1 levels in a dose-dependent manner. Moreover, it has been reported that LPA elicits transient activation of *Rho*A, which is an essential event in cell migration [20, 21]. However, the cellular effects of cPA, an LPA analog, are remarkably different from those of LPA. cPA inhibits cell proliferation [22], induces actin stress fiber formation [23], inhibits LPA-induced platelet aggregation [24], and inhibits cancer cell invasion and metastasis [25].

**Figure 3:**
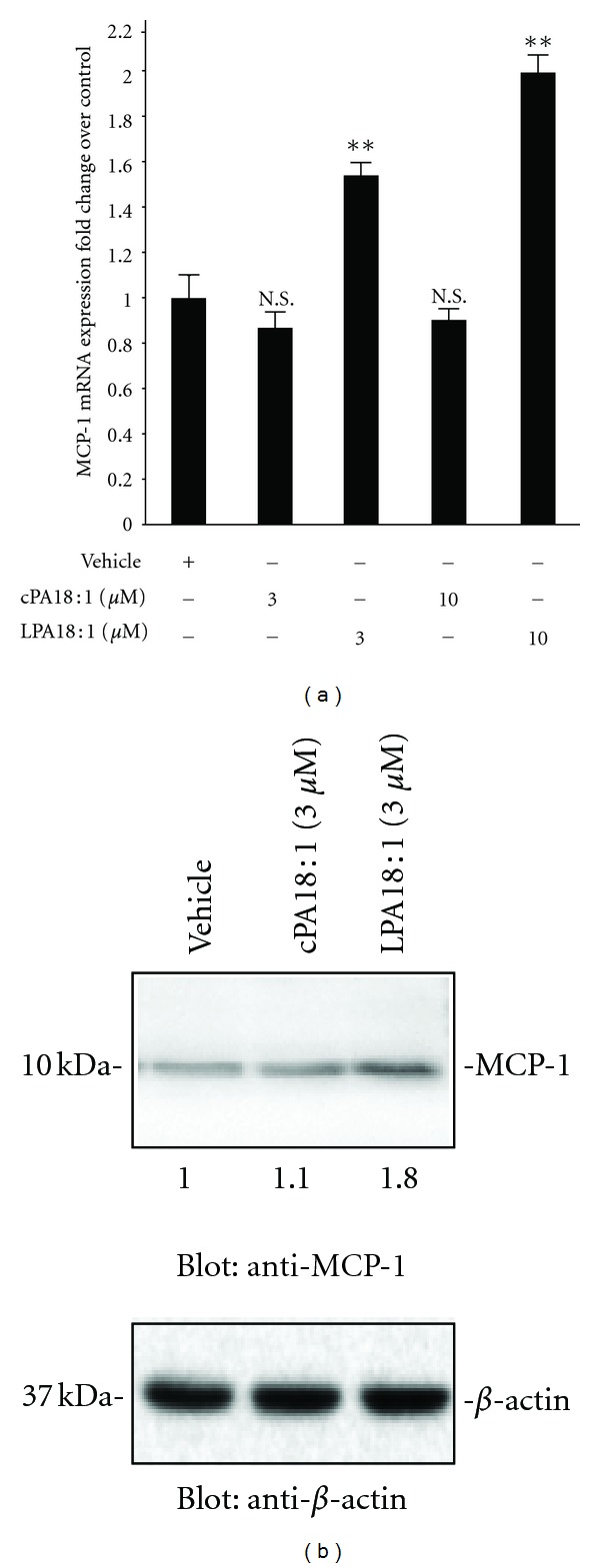
LPA induction of the secretion of MCP-1 was confirmed by RT-PCR and western blot analysis. (a) C2C12 cells were exposed to vehicle, unsaturated forms of LPA (18 : 1) (3 *μ*M), and cPA (18 : 1) (3 *μ*M) for 24 h, and RNA was isolated. The mRNA levels of MCP-1 were determined by real-time PCR. MCP-1 mRNA levels were normalized to 18S rRNA (mean ± SEM, *n* = 3; **P < 0.01 by ANOVA test). (b) Western blot analysis of MCP-1 protein secreted in the conditioned media of C2C12 cells stimulated by LPA. Starved cells were treated with LPA or cPA for 24 h. The conditioned media were harvested and examined by western blot analysis, after exposure of C2C12 cells to vehicle. *β*-Actin was used as a loading control.

**Figure 4:**
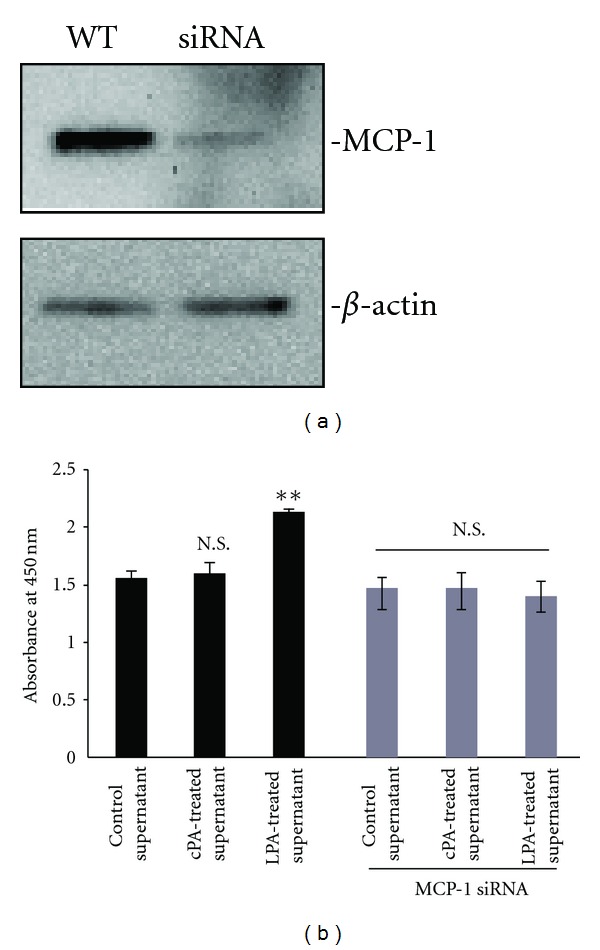
MCP-1 stimulates cell proliferation through paracrine pathways. (a) siRNA-mediated MCP-1 knockdown. Levels of MCP-1 in cells transfected with siRNA against MCP-1 for 24 h were determined by western blot analysis. (b) Wild-type C2C12 cells and MCP-1 siRNA-transfected C2C12 cells were cultured with LPA (10 *μ*M), cPA (10 *μ*M), or vehicle (DMSO) for 24 h. The conditioned media were added to the each well, and cells were incubated for 24 h. Cell proliferation was determined with Cell Counting Kit-8. Ten microliters of the reagent was added to the medium, and cells were incubated for 2 h in a 5% CO_2_ incubator. Data are presented as mean ± SEM (*n* = 3). **P < 0.01 by ANOVA test.

To substantiate the array findings on LPA-stimulated chemokine secretion, we measured MCP-1 protein secretion in the conditioned medium by western blot analysis. As shown in Figure 3(a), real-time PCR analysis revealed that MCP-1 mRNA expression was increased (2.0-fold) after exposure to 10 *μ*M LPA for 24 h. The conditioned media were collected and concentrated with a centrifugal filter device (Amicon Ultra, 3 kDa). We then investigated the specificity of MCP-1 antibody in the conditioned medium from C2C12 cells by western blotting, after exposure to LPA or cPA (Figure 3(b)). MCP-1 protein expression was enhanced by LPA and increased 1.8-fold compared with vehicle-treated cells. We next determined whether MCP-1 could play a regulatory role in cell proliferation after exposure to LPA. We assessed the MCP-1 production by incubating the conditioned medium with LPA and then treated the C2C12 cells or MCP-1 siRNA-transfected C2C12 cells with the medium harvested from LPA- or cPA-exposed cells or with control [dimethyl sulfoxide (DMSO)] conditioned medium. As shown in Figure 4(b), LPA-treated cells showed enhanced cell proliferation compared with cPA or vehicle control. However, MCP-1 siRNA-transfected C2C12 cells showed no increase in proliferation after exposure to LPA. These data suggest that LPA directly acts on MCP-1 expression and secretion in C2C12 myoblasts. In addition, these results demonstrate the paracrine role of LPA in regulating the proliferation of C2C12 cells. Taken together, our data clearly show that LPA, but not cPA, induces a concentration-dependent secretion of MCP-1 from C2C12 cells. This study is the first to report an increase in the levels of MCP-1 and its mRNA in skeletal muscle C2C12 cells after exposure to LPA. Our present study revealed the molecular mechanisms of LPA induction of MCP-1 secretion in myoblasts, and thus it provides important information regarding inflammatory conditions, including RA. MCP-1 has a key role in conditions ranging from acute respiratory distress syndrome to rheumatoid arthritis [26]. Our findings indicate that the MCP-1 chemokine plays an important role in LPA-mediated myoblast proliferation. Oxidized LDL (moxLDL) particles stimulate MCP-1 synthesis by vascular cells [27]. Zhang et al. reported that moxLDL leads to the generation of LPA and elicits pleiotropic growth-factor-like effects on most cell types [28]. Taken together with the finding that siRNA-mediated silencing of MCP-1 abrogated the LPA-induced C2C12 cell proliferation, these results lead us to suggest that molecular mechanisms of LPA induction of MCP-1 secretion in C2C12 cells provides valuable information that may contribute to the myoblast cell differentiation.
